# Nonpareil 3: Fast Estimation of Metagenomic Coverage and Sequence Diversity

**DOI:** 10.1128/mSystems.00039-18

**Published:** 2018-04-10

**Authors:** Luis M. Rodriguez-R, Santosh Gunturu, James M. Tiedje, James R. Cole, Konstantinos T. Konstantinidis

**Affiliations:** a School of Civil and Environmental Engineering, Georgia Institute of Technology, Atlanta, Georgia, USA; b School of the Biological Sciences, Georgia Institute of Technology, Atlanta, Georgia, USA; c Center for Microbial Ecology, Michigan State University, East Lansing, Michigan, USA; d Department of Microbiology and Molecular Genetics, Michigan State University, East Lansing, Michigan, USA; e Department of Plant, Soil and Microbial Sciences, Michigan State University, East Lansing, Michigan, USA; University of North Carolina at Charlotte

**Keywords:** bioinformatics, coverage, metagenomics, microbial ecology

## Abstract

Estimations of microbial community diversity based on metagenomic data sets are affected, often to an unknown degree, by biases derived from insufficient coverage and reference database-dependent estimations of diversity. For instance, the completeness of reference databases cannot be generally estimated since it depends on the extant diversity sampled to date, which, with the exception of a few habitats such as the human gut, remains severely undersampled. Further, estimation of the degree of coverage of a microbial community by a metagenomic data set is prohibitively time-consuming for large data sets, and coverage values may not be directly comparable between data sets obtained with different sequencing technologies. Here, we extend Nonpareil, a database-independent tool for the estimation of coverage in metagenomic data sets, to a high-performance computing implementation that scales up to hundreds of cores and includes, in addition, a *k*-mer-based estimation as sensitive as the original alignment-based version but about three hundred times as fast. Further, we propose a metric of sequence diversity (*N*_*d*_) derived directly from Nonpareil curves that correlates well with alpha diversity assessed by traditional metrics. We use this metric in different experiments demonstrating the correlation with the Shannon index estimated on 16S rRNA gene profiles and show that *N*_*d*_ additionally reveals seasonal patterns in marine samples that are not captured by the Shannon index and more precise rankings of the magnitude of diversity of microbial communities in different habitats. Therefore, the new version of Nonpareil, called Nonpareil 3, advances the toolbox for metagenomic analyses of microbiomes.

**IMPORTANCE** Estimation of the coverage provided by a metagenomic data set, i.e., what fraction of the microbial community was sampled by DNA sequencing, represents an essential first step of every culture-independent genomic study that aims to robustly assess the sequence diversity present in a sample. However, estimation of coverage remains elusive because of several technical limitations associated with high computational requirements and limiting statistical approaches to quantify diversity. Here we described Nonpareil 3, a new bioinformatics algorithm that circumvents several of these limitations and thus can facilitate culture-independent studies in clinical or environmental settings, independent of the sequencing platform employed. In addition, we present a new metric of sequence diversity based on rarefied coverage and demonstrate its use in communities from diverse ecosystems.

## Abstract (precis)

Estimation of the coverage provided by a metagenomic data set, i.e., what fraction of the microbial community was sampled by DNA sequencing, represents an essential first step of every culture-independent genomic study that aims to robustly assess the sequence diversity present in a sample. However, estimation of coverage remains elusive because of several technical limitations associated with high computational requirements and limiting statistical approaches to quantify diversity. Here we described Nonpareil 3, a new bioinformatics algorithm that circumvents several of these limitations and thus can facilitate culture-independent studies in clinical or environmental settings, independent of the sequencing platform employed. In addition, we present a new metric of sequence diversity based on rarefied coverage and demonstrate its use in communities from diverse ecosystems.

## INTRODUCTION

The exploration of microbial diversity in natural and engineered environments has been revolutionized by the use of metagenomics. However, the power of both descriptive and comparative metagenomic analyses is strongly deterred by low coverage, defined as the fraction of the DNA space covered by sequencing (1). To date, most metagenomic studies assess the level of coverage only indirectly or not at all, mainly owing to the difficulty of directly measuring the unseen fraction of a community. For instance, many studies have assessed coverage by extracting 16S rRNA gene-containing reads from amplicon or shotgun metagenomes, clustering these sequences in operational taxonomic units (OTUs) on the basis of their best matches in reference databases or by *de novo* clustering, and counting the discovery of new OTUs with an increasing number of available sequences. The degree to which this approach represents the real diversity of the sample remains essentially unknown because it depends on the unbiased recovery of 16S rRNA gene-containing reads from the metagenome, the comprehensiveness of the reference database in representing the natural diversity in the sample, the extent of undersampling of the diversity, and the fact that identical 16S rRNA gene sequences may represent distinct species because of the high degree of sequence conservation of the rRNA genes (2).

We have recently presented a method to assess the level of coverage provided by metagenomic data sets by a redundancy-based approach, Nonpareil (3). Briefly, Nonpareil estimates the read redundancy in a metagenomic data set by using ungapped alignments of a subset of sequencing reads against the entire metagenome and then applies the Turing-Good estimator principle to approximate the abundance-weighted coverage of the metagenome. Abundance weighting means that the coverage estimate represents the fraction of the total DNA extracted from a sample (and by extension, the fraction of the organisms, not species) that is represented in a set of metagenomic sequence data, not necessarily how many different species are represented by the extracted versus the sequenced DNA. For instance, the nonsequenced fraction may represent a higher number of species even in cases where the coverage is >50% if the community is characterized by high species evenness, which is not uncommon for soil and sediment habitats. Nonpareil is database independent because it is based on intrinsic characteristics of the data set and, given sufficient data (typically, a small fraction of most available metagenomes), can produce robust estimations of coverage regardless of the sequencing effort applied.

Two previously described techniques for the estimation of coverage in metagenomes exist. The first one, COVER (4), assumes reference database completeness and a high degree of taxonomic conservation in 16S rRNA gene copy number and genome size, assumptions often violated by available genome sequences (5). Accordingly, Nonpareil is substantially more accurate than COVER for most complex communities as previously shown (3). The second one is a parametric approach currently not implemented in any available standalone tool (6) that models occupancy as a Poisson process using a gamma approximation and requires estimates of abundance and genome size joint distributions. Other approaches related to the problem of coverage exist but offer only indirect measurements such as the maximum expected contig size (7) or the coverage of a target minimum-abundance organism (8, 9) from which only an implementation for viral communities exists, i.e., MetLab (10). Therefore, Nonpareil is advantageous compared to previously described approaches and bioinformatic tools in that it is database independent, directly estimates coverage, and is fully automated, allowing the end user to simply input a metagenomic data set for coverage estimation. In addition, Nonpareil subsamples the estimated coverage and fits the rarefied curve to a sigmoidal function in order to predict the sequencing effort necessary to reach any target coverage (3). Using Nonpareil, we have shown that coverage affects not only the completeness of descriptive metagenome-based profiling but also the accuracy of comparative abundance analyses of features such as species or genes, highlighting the importance of coverage estimations for both descriptive and comparative metagenomics (1).

While we have previously demonstrated that Nonpareil accurately estimates the level of coverage in less complex metagenomic data sets, the estimation remained prohibitively expensive for data sets comprising several billion base pairs. Here, we present a new version of Nonpareil, Nonpareil 3, that effectively distributes the estimation across processors and computing nodes by using high-performance computing capabilities now often available to laboratories working with metagenomic data. Nonpareil 3 also includes a new algorithm providing an alternative estimation of coverage based on *k*-mer redundancy, as opposed to ungapped alignment in the original version, with comparable accuracy but 2 orders of magnitude faster computation. This version allows end users to run Nonpareil analysis of relatively large metagenomic data sets on personal computers as well. Moreover, we previously showcased the qualitative sequence diversity rankings derived from Nonpareil curves (1, 3). Here, we quantitatively assessed the level of sequence diversity derived from Nonpareil curves, compared these estimations with other diversity indices, and present typical ranges for communities from different environments, allowing for quantitative ranking of the communities on the basis of their sequence complexity.

## RESULTS

### Reducing run time for large metagenomes.

**FIG 1:**
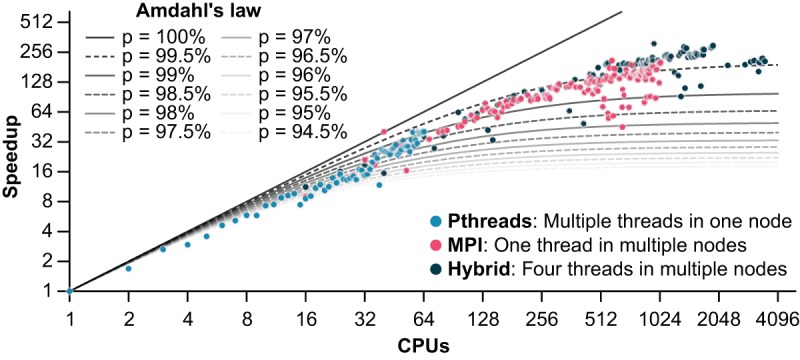
Speedup per number of processors in Nonpareil. Nonpareil estimations of the LL_1007B data set (3) were performed with alignment kernel and default parameters in multiple processors of a node (light blue), a single processor of multiple nodes (pink), and four processors of multiple nodes (dark green). The base time (one processor of one node) was 82.98 h.

Nonpareil 3 can use parallelization across nodes and threads by using Message Passing Interface (MPI) and pthreads, respectively. We executed Nonpareil with the original alignment kernel by using both parallelization methods, independently and in combination, on a 2.3-Gbp test data set in order to evaluate the speedup. These optimizations resulted in up to 500 times faster computation of coverage. Compared to Amdahl’s law, Nonpareil 3 speedups corresponded to around 99.5% parallelization with MPI alone and around 99.8% parallelization with MPI and four threads, while the observed speedup for multithreads in one node was essentially linear (Fig. 1). In addition, the read redundancy can now be estimated by using perfect matches of one *k*-mer per query read corrected by sequencing error estimation, instead of the complete ungapped alignment. This implementation results in similar coverage and diversity estimates, as well as highly correlated projections of sequencing effort, but reduces the computational time by about 300 times (Table 1; see Fig. S1 in the supplemental material). We recommend using *k* = 24 (the default value in Nonpareil), the smaller value producing stable estimates (Fig. S2). The runtime of Nonpareil with alignment kernel is approximately proportional to *N* × *Q* × *L*^2^, where *N* is the total number of sequencing reads, *Q* is the number of query sequencing reads searched against the complete metagenomic data set, and *L* is the length of a read. In contrast, the complexity of Nonpareil with *k*-mer kernel is simply *N* × *L*, i.e., directly proportional to the total data set size (Fig. S3). As the runtime for the *k*-mer kernel is independent of the read length, Nonpareil 3 is directly compatible with long-read data, although current technologies (e.g., PacBio or MinION) exhibit error rates that are higher than optimal for the current Nonpareil implementation (i.e., <5% expected sequencing error). Thus, additional optimizations will be necessary to allow reads with higher sequencing error to be analyzed by Nonpareil. In addition, since the runtime is not proportional to *Q*, it is practical to use a larger value for *Q* (default of 10,000 instead of 1,000 in the alignment kernel), increasing the precision of Nonpareil over that of the original version.

10.1128/mSystems.00039-18.1 FIG S1  Nonpareil curves for data sets highlighted in this study using *k*-mer and alignment kernels. (A to G) The Nonpareil curves show the fit of coverage per sequencing effort to a sigmoidal model. The lines indicate coverage estimates from subsampling (solid) and Nonpareil projection curves (dashed), and the lower-end arrows indicate the sequence diversity. Horizontal red dashed lines indicate 95 and 99% coverage. Colors indicate the kernel and parameters used, with the *k*-mer kernel in magenta, the alignment kernel with 95% identity in blue, and the alignment kernel with 99% identity in gray. (H) *N*_*d*_ for the curves in panels A to G. Download FIG S1, PDF file, 0.6 MB. Copyright © 2018 Rodriguez-R et al. 2018 Rodriguez-R et al. This content is distributed under the terms of the Creative Commons Attribution 4.0 International license.

10.1128/mSystems.00039-18.2 FIG S2  Stability of *k*-mer lengths between 18 and 32. Estimated abundance-weighted average coverage (A), sequence diversity (B), and projected sequencing effort to reach 95% coverage (C) for seven data sets using *k*-mer lengths between 10 and 32. Note that most estimates stabilize at a length of 18 onward and some estimates of coverage stabilize at a length of 24. The default value in Nonpareil (*k* = 24) is indicated by black vertical bars. Download FIG S2, PDF file, 0.3 MB. Copyright © 2018 Rodriguez-R et al. 2018 Rodriguez-R et al. This content is distributed under the terms of the Creative Commons Attribution 4.0 International license.

10.1128/mSystems.00039-18.3 FIG S3  Linear complexity of Nonpareil with *k*-mer and alignment kernels. Data set size and running times are from Table S1, showing a linear time increase with input size. Download FIG S3, PDF file, 0.1 MB. Copyright © 2018 Rodriguez-R et al. 2018 Rodriguez-R et al. This content is distributed under the terms of the Creative Commons Attribution 4.0 International license.

10.1128/mSystems.00039-18.7 TABLE S1  Genomes used for simulated data sets to evaluate error correction. Download TABLE S1, PDF file, 0.04 MB. Copyright © 2018 Rodriguez-R et al. 2018 Rodriguez-R et al. This content is distributed under the terms of the Creative Commons Attribution 4.0 International license.

**TABLE 1:** Kernel comparison of Nonpareil estimates for publicly available data sets^a^

**Sample**	**Identifier**	**Reference**	**Size (Gbp)**	**CPU time (min)**		**% coverage**		**Required effort (Gbp)**	
				A	K	A	K	A	K
Posterior fornix	SRS063417	25	0.01	15.7	0.08	89	84	0.062	0.070
Stool sample	SRS015540	25	0.32	438	0.85	81	71	2.62	5.55
Tongue	SRS055495	25	0.22	286	0.68	71	61	3.22	6.08
LL 2011	SRR948155	3	2.95	4,397	16.5	84	79	11.7	24.1
LL 2009A	SRR096386	26	1.17	1,444	6.40	68	64	20.5	24.8
LL 2009B	SRR096387	26	1.12	1,463	5.75	70	64	14.3	20.0
Iowa soil	JGI 402461	NA^b^	14.6	22,806^c^	49.0	56	48	662	1,051

a The two kernels (A, alignment; K, *k*-mer) were compared in terms of CPU time, estimated coverage, and projected required sequencing effort to reach 95% coverage of samples varying in complexity, including HMP (posterior fornix, tongue, stool sample), freshwater (Lake Lanier [LL]), and soil (Iowa continuous cornfield).

b NA, not available.

c CPU time was estimated for Iowa soil and observed in all other cases.

### Sequencing error correction.

In order to reduce the impact of sequencing error on Nonpareil with *k*-mer kernel, we implemented a redundancy correction based on the sequencing read quality scores. Briefly, we estimated the fraction of selected *k*-mers that are expected to include at least one sequencing error from the quality scores and removed that fraction from the list of *k*-mers without matches, assuming that the introduced errors would most frequently result in unobserved variants (see Materials and Methods for details). To test this correction, low- and high-coverage simulated metagenome-like data sets of 101-bp reads (507,813 and 7,093,697 reads, respectively) were generated *in silico* from 30 bacterial and archaeal genomes by a previously described method (3) (Table S1; data sets are available at http://enve-omics.ce.gatech.edu/data/nonpareil). For each test, 10,000 reads were randomly selected from the simulated data sets and modified with substitution errors at a constant error probability per base. The 10,000 reads were then used in Nonpareil with *k*-mer kernel as query sequences with error correction enabled and disabled (Table S2; Fig. S4). In general, error rates affected the estimation of both coverage and required sequencing effort when error correction was disabled but not when it was enabled, indicating that the correction effectively removes the effect of sequencing errors when the error probability estimation is accurate.

10.1128/mSystems.00039-18.4 FIG S4  Effect of error correction in *k*-mer kernel. Nonpareil curves for *k*-mer kernel with and without error correction are shown. A, low (48%) coverage; B, high (98%) coverage. Note that the expected optimal result (0% error) overlaps all of the data sets with error correction regardless of the amount of error (shades of fuchsia), while Nonpareil curves and sequence diversity estimates deviated significantly when error was incorporated without applying the error correction function (shades of gray). Download FIG S4, PDF file, 0.3 MB. Copyright © 2018 Rodriguez-R et al. 2018 Rodriguez-R et al. This content is distributed under the terms of the Creative Commons Attribution 4.0 International license.

10.1128/mSystems.00039-18.8 TABLE S2  Nonpareil error correction for *k*-mer kernel. Download TABLE S2, PDF file, 0.05 MB. Copyright © 2018 Rodriguez-R et al. 2018 Rodriguez-R et al. This content is distributed under the terms of the Creative Commons Attribution 4.0 International license.

### Nonpareil *N*_*d*_.

The Nonpareil index of sequence diversity (*N*_*d*_), described in Materials and Methods, is expressed in units of the natural logarithm of base pairs and summarizes the community diversity in sequence space, i.e., how redundant (or conversely unique) the sequences of a data set are among themselves. This metric depends on the joint distribution of genome size and abundance, as well as intragenome gene duplication. Therefore, given a small variation in genome size and a small impact of genomic duplications, e.g., for prokaryote-only communities, *N*_*d*_ can be used as a database-independent metric of alpha diversity. Since the shapes of the Nonpareil curves from replicates and subsamples closely resemble each other regardless of coverage (3), we propose *N*_*d*_ as a coverage-independent measurement of the diversity of the sampled community (Fig. S5).

10.1128/mSystems.00039-18.5 FIG S5  Coverage-independent projection and sequence diversity. (A) Nonpareil curves for a reference data set (LL-autumn; black) and three random subsamples at each of the labeled fractions (different colors). Note that the projections of all of the subsamples overlap. Sequence diversity (B) and projected sequencing effort (*LR*) required to reach 95% coverage (C) by estimated coverage of the reference sample and random subsamples. All data sets (reference and subsamples) are available at http://enve-omics.ce.gatech.edu/data/nonpareil. Download FIG S5, PDF file, 0.5 MB. Copyright © 2018 Rodriguez-R et al. 2018 Rodriguez-R et al. This content is distributed under the terms of the Creative Commons Attribution 4.0 International license.

**FIG 2:**
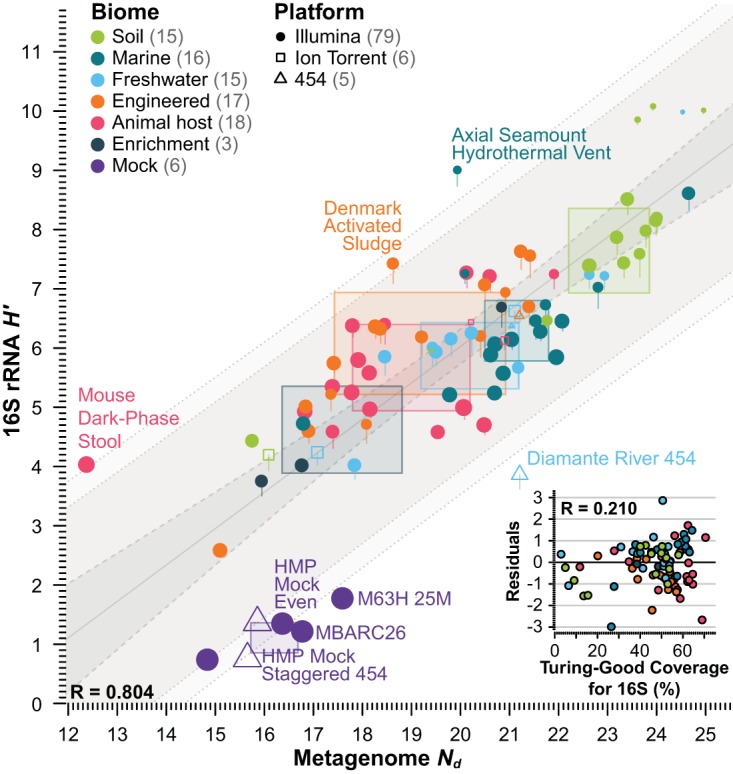
Comparison of Nonpareil *N*_*d*_ sequence diversity and 16S rRNA gene OTU Shannon *H′* taxonomic diversity indices on 90 metagenomes. Each data point represents the estimates on *N*_*d*_ (*x* axis) and *H′* (*y* axis). The *y*-axis value of each point indicates the Bayesian analysis-corrected Shannon index, and the line extending from the low part of each data point represents the exact observed (maximum-likelihood) Shannon index. The color of each point indicates the type of biome of each data set, the shape indicates the sequencing platform, and the size indicates the estimated coverage of the 16S rRNA gene profile (Turing-Good estimate). For each biome, the IQR of both estimates is represented as semitransparent rectangles. The least-squares linear correlation model is represented in gray, including the central estimate (solid line), the 95% confidence interval (dashed-line band), and the 80 and 95% prediction intervals (dotted-line bands). Labeled data sets fell outside the 80% prediction interval. The inset shows the residuals from the linear model against the Turing-Good estimate of 16S rRNA gene coverage.

We compared *N*_*d*_ and Shannon diversity index values in natural units (*H′*) from 16S rRNA gene OTU tables in different collections of metagenomic data sets, including: set I, metagenomes from different biomes including six mock data sets; set II, metagenomes and 16S rRNA gene amplicons from human microbiomes of different body sites; and set III, a collection of marine metagenomes from different sampling sites. First, we observed a high correlation (Pearson’s *r* = 0.804 [*P* < 10^−15^]; analysis of variance [ANOVA], 64.6% variance explained [*P* < 10^−15^]) between *N*_*d*_ and *H′* values from diverse environments (set I), as well as a monotonic trend for interquartile ranges (IQRs) between different biomes (Fig. 2). This correlation was maintained in the subset of mock samples alone (*r* = 0.87 [*P* = 0.023]), and the residuals were only slightly affected by sequencing technology (ANOVA after including *N*_*d*_, 3.7% variance [*P* = 0.008]). However, most outliers had low Turing-Good coverage estimates for the 16S rRNA gene OTU count profiles (labeled points in Fig. 2). Indeed, we observed a significant effect of the 16S rRNA gene Turing-Good coverage estimates on the residuals (ANOVA after *N*_*d*_, excluding mock samples, 5.7% variance [*P* = 10^−4^]; ANOVA after *N*_*d*_, including mock samples assuming complete coverage, 14.7% variance [*P* = 10^−11^]) (Fig. 2, inset). We did not observe a significant effect of the Nonpareil-estimated coverage on the model residuals (ANOVA after *N*_*d*_, 0.008% variance [*P* = 0.9]), indicating that a significant component of the difference between the two estimates of diversity was due to insufficient data to robustly estimate *H′* but not *N*_*d*_. We were able to differentiate between the effect of coverage on both estimates independently because the correlation between 16S rRNA gene-derived Turing-Good coverage estimates and metagenome-derived Nonpareil coverage estimates was partially lost by subsampling of most metagenomic data sets in the estimation of *N*_*d*_ but not in the estimation of *H′* (*r* = 0.64). (Note that *H′* was estimated on the basis of previously constructed OTU tables on the complete data sets available [11], not subsamples as in the case of *N*_*d*_; see Materials and Methods for details.)

Next, we evaluated paired metagenomic and 16S rRNA gene amplicon samples from the Human Microbiome Project (HMP) (set II) and observed a similarly high correlation between median *N*_*d*_ and median *H′* values per body site (*r* = 0.93), as well as between *N*_*d*_ and *H′* values per data set (*r* = 0.84; Fig. S6).

10.1128/mSystems.00039-18.6 FIG S6  Comparison of Nonpareil *N*_*d*_ sequence diversity and amplicon 16S rRNA gene OTU Shannon *H*′ diversity indices for HMP data sets. (A) *N*_*d*_ of metagenomes and *H*′ of 16S rRNA gene amplicons for 13 body sites from the HMP, where circles represent the median, whiskers represent the 90% central range, and the numbers of metagenomic and 16S rRNA gene data sets used are indicated in parentheses for each site, respectively. (B) *N*_*d*_ of metagenomes and *H*′ of 16S rRNA gene amplicons for 53 samples including both types of data sets in the HMP collection. Colors indicate the sampling body site as in panel A. In both panels, the least-squares linear correlation model is represented by a black line and the 95% confidence interval is indicated by a gray band. Download FIG S6, PDF file, 0.2 MB. Copyright © 2018 Rodriguez-R et al. 2018 Rodriguez-R et al. This content is distributed under the terms of the Creative Commons Attribution 4.0 International license.

Finally, we explored a collection of marine data sets (set III) and compared *N*_*d*_ and *H′* diversity indices against available metadata by using ANOVA with alpha = 0.01. After controlling for the variance introduced by the source project (as the first independent variable in ANOVAs), we evaluated the effects of different variables on diversity variation (see Materials and Methods). For both measurements of diversity (*N*_*d*_ and *H′*), we observed significant effects of size fraction (*H′* variance explained, 16%; *N*_*d*_ variance explained, 25% [*P* < 10^−11^]) and latitude (*H′* variance explained, 6.7%; *N*_*d*_ variance explained, 1.5% [*P* < 0.01]), similar to previously reported results for estimated richness as the measure of alpha diversity (12). Geographic location was also found to significantly affect the variance of *N*_*d*_ (12% variance, *P* = 10^−8^) and much more weakly so the variance of *H′* (4.3% variance, *P* = 0.02). Moreover, *N*_*d*_ captured a pattern of increased diversity toward the winter (wintriness; explained variance, 2.3% [*P* = 0.0011]) not observed with *H′* (explained variance, 0.14% [*P* = 0.46]).

## DISCUSSION

Estimations of coverage from sequencing data have been a problem studied since the onset of DNA sequencing techniques (reviewed in reference 13). However, most efforts have been directed at estimation of the coverage of a single genome such as those proposed early on by Lander and Waterman (14), as well as more refined models later proposed by Wendl and collaborators (8, 9, 15). Few attempts have been made to extend some of these concepts to entire communities assuming joint distributions for abundance, genome size, and other relevant genomic features (6, 7). However, such attempts have only indirectly addressed the problem of coverage, ultimately targeting other characteristics such as the maximum expected contig length. Therefore, these approaches are not directly comparable to the Nonpareil estimate of coverage. Intrinsic characteristics of random samplings (such as metagenomic data sets) can also be leveraged to determine the level of coverage by using the principle of the Turing-Good estimator (16, 17). This principle has been applied to the estimation of diversity in OTU profiles (18, 19), and similar approaches have been explored for the extrapolation of any count statistics derived from sequencing data (20). Nonpareil uniquely uses this principle to directly estimate the abundance-weighted average coverage of a metagenomic data set based on the degree of overlap of individual metagenomic reads (3).

We have implemented algorithmic and computational improvements in Nonpareil 3 that now make it feasible to process large metagenomic data sets (e.g., tens to hundreds of gigabase pairs) in minutes to hours, even with modest computational resources and without compromising accuracy. Moreover, in addition to the estimation of abundance-weighted average coverage and the projection to estimate required sequencing efforts, Nonpareil 3 includes an estimation of sequence diversity, *N*_*d*_. We demonstrate that *N*_*d*_ correlates well with alpha diversity derived from 16S rRNA gene OTU profiles but can also capture patterns in diversity not observed with 16S rRNA gene profiles, likely because of the increased taxonomic resolution attainable by whole-genome analyses. For example, seasonal diversity patterns observed with *N*_*d*_ in marine metagenomic data sets were not captured by 16S rRNA gene OTU diversity measured by Shannon *H′*. Therefore, Nonpareil advances the molecular toolbox for environmental surveys, providing estimations of coverage and diversity independent of databases and robust to various levels of sequencing effort applied, required sequencing effort for complete coverage, and sequence diversity, and scales well with large metagenomic data sets. Applying Nonpareil to metagenomic data sets from different biomes allowed us to quantify sequence diversity and its typical range for different environments. Unsurprisingly, the most diverse communities were those in soil, with an *N*_*d*_ IQR of 22 to 24. Marine communities (open ocean) followed soil communities at about 2 units lower, with an *N*_*d*_ IQR of 20.5 to 21.8. Because of the logarithmic nature of *N*_*d*_, this corresponds to a sequence diversity about seven times lower in marine than in soil communities. We observed wider and largely overlapping ranges of *N*_*d*_ in communities from freshwater (IQR, 19.5 to 21.1), engineered systems (IQR, 17.8 to 20.7), and animal hosts (IQR, 18.1 to 20.3), all of which are about 2 to 7 times less diverse than those in the open ocean and about 15 to 45 times less diverse than those in soil. Importantly, these differences in sequence diversity translate to differences of several orders of magnitude in required sequencing effort in order to achieve a similar level of coverage (3). Human-associated communities (*n* = 8) did not differ significantly from nonhuman animal-associated samples (*N*_*d*_ IQR, 18.2 to 20.1 versus 17.6 to 20.3), including 4 mouse samples, 2 cow samples, 2 pig samples, 1 chicken sample, and 1 salmon sample (*n* = 10). These results should represent useful reference points for designing future metagenomic studies and achieving the level of coverage that would be desirable for each project and its research objective(s).

## MATERIALS AND METHODS

### Implementation.

The processing of a sample in Nonpareil is divided into two main steps. The first is redundancy estimation, where sequences are compared and the estimated redundancy is subsampled at different values of sequencing effort. The large number of pairwise alignments makes this step the most resource intensive. This task is now distributed across multiple nodes using MPI and processors using C++ pthreads. Further, Nonpareil 3 offers a *k*-mer-based method for redundancy estimation (see below) that accelerates this step by using short fragments of the sequencing reads with no errors allowed. Finally, redundancies are subsampled in multiple threads. In Nonpareil 1, subsamples were estimated linearly, resulting in sparse values toward the left side of the Nonpareil curve. Although this strategy is still available, the default in Nonpareil 3 is logarithmic subsampling; sample size is iteratively multiplied by a density factor (default 0.7) until only two reads remain.

The second step is estimation of the abundance-weighted average coverage at different sequencing efforts (Nonpareil curves), fitting to a sigmoidal model (projection), and graphical representation (e.g., Fig. S1). Note that this step relies on the assumption of independence of events between sequencing reads; therefore, Nonpareil should be applied on single reads, one of the sister reads in paired-end reads, or merged paired-end reads (3). This step has modest resource requirements and is implemented in the Nonpareil R package. In Nonpareil 3, we have streamlined this analysis by using headers in the redundancy output files and included an estimation of the sequence diversity derived from the fitted model (see below). All of the experiments in this report were executed with Nonpareil v3.3.

### *k*-mer kernel*.*

To accelerate read-to-read comparisons, Nonpareil 3 now implements a *k*-mer-based comparison kernel. Briefly, query *k*-mers are derived from a randomly selected subset of the metagenomic data set reads, with one target *k*-mer selected from the 5′ end of each read. Determination of the number of times each target *k*-mer (or its reverse complement) is found at any position of any sequence read in the complete data set can be performed in time proportional to the size of the metagenomic data set and is independent of the number of target *k*-mers. Note that this test is unable to detect if any of the last *k* − 1 bases in a sequencing read cover the target *k*-mer, so for each metagenomic read of length *L*, only *L* − *k +* 1 positions can be tested for matches, reducing the effective size of the metagenomic data set scanned with respect to the complete alignment kernel. Nonpareil curves (R package) account for the effect of this difference between sequencing effort and effective size, and its effect is corrected.

Because the target *k*-mers are derived from the metagenomic sequence reads, some of the *k*-mers will contain sequence errors, but if *k* is large enough, these *k*-mers will likely have zero coverage. Although it is not possible to determine which of the zero-coverage target *k*-mers contain errors, we utilized the sequencing quality (*Q*) scores from each target *k*-mer’s parent sequencing read to estimate *E*, the expected number of *k*-mers with errors. To correct for these errors, we reduced the target set by removing *E* zero-coverage target *k*-mers. The coverage counts for the remaining target *k*-mers, along with the reduced effective metagenomic data set size, are passed through the remaining original Nonpareil steps to estimate coverage, required sequencing effort, and sequence diversity.

### *k*-mer kernel comparison to the original alignment-based kernel.

To compare the results obtained with the traditional alignment kernel and the novel *k*-mer kernel, we executed both analyses in seven metagenomic data sets with different degrees of diversity (Table 1; Fig. S1). Metagenomic data sets were processed by using SolexaQA (21) with a maximum expected error of 1% and a minimum length of 50 bp, and adapter contamination was clipped by using Scythe (https://github.com/vsbuffalo/scythe). For paired-end samples, only the forward reads were used. Short Read Archive (SRA) identifiers are provided in Table 1 for all of the data sets except Iowa continuous cornfield soil. For the latter, seven lanes from one run of Illumina HiSeq were retrieved from the JGI Genome Portal (http://genome.jgi.doe.gov) on 21 July 2016 from project 402461.

Nonpareil results with *k*-mer kernel were obtained by using a 27-in. iMac with 8 gigabytes of random-access memory and an Intel Core i5 3.2-GHz processor using *k* = 24, 10,000 queries, and two threads (only for subsampling, the *k*-mer matching portion of Nonpareil is single threaded). Nonpareil alignment kernel results for Iowa soil were obtained by using the Michigan State University High-Performance Computing Center nodes with 20 cores and the following options: 20 threads, 1,000 queries, 50% overlap, and a redundancy to coverage transformation factor of 1.0. All other data sets were processed with a 27-in. iMac as described above and with the same Nonpareil alignment options at 95% identity and two threads. Iowa soil central processing unit (CPU) time for alignment kernel was estimated by using the linear regression of the other data sets.

### *N*_*d*_.

Nonpareil curves are plots of abundance-weighted average coverage (*Ĉ*) per sequencing effort (*LR*) fitted to the cumulative probability function of the gamma distribution (3) with parameters α and β as follows: *Ĉ* = γ[α, β × log(*LR* + 1)]/Γ(α), where Γ is the gamma function and γ is the lower incomplete gamma function. Hence, we can use the mode of the corresponding gamma distribution to identify the value of log(*LR* + 1) corresponding to the inflection point of the curve, which we propose as a measurement of sequence diversity as follows: *N*_*d*_ *=* (α − 1)/β.

To evaluate the correlation between *N*_*d*_ and traditional measurements of alpha diversity derived from taxonomic affiliation, we compiled three collections of metagenomic data sets. Set I consisted of 90 samples from multiple environments, set II consisted of 54 human-associated microbiome metagenomic samples, and set III consisted of 292 marine samples. Set I was used to evaluate the general agreement between traditional taxonomy-based alpha diversity and *N*_*d*_. It consisted of 84 metagenomic data sets from multiple environments divided into six distinct biomes plus six mock data sets (Table S3). Subsamples of processed nucleotide reads (between one and seven file chunks of 0.5 gigabyte zipped, as necessary to surpass 60% coverage or as many files as available) and OTU tables based on metagenome-derived 16S rRNA gene-containing reads were obtained from EBI Metagenomics (11). The Shannon index (*H′*) of each OTU table was estimated on the basis of Bayesian estimates of frequencies by using the Dirichlet multinomial pseudocount model with Laplace prior, as implemented in the R package entropy (22), with the exception of mock samples, for which maximum-likelihood entropy of input concentrations was used. Note that the 16S rRNA gene-containing reads derived from metagenomes do not necessarily overlap; hence, the EBI Metagenomics Pipeline uses closed-reference OTU picking (23), potentially biasing the results by database completeness. Therefore, we extended this analysis by using set II derived from the HMP (24). This collection consisted of samples from 13 body sites including 54 metagenomic data sets and 3,613 16S rRNA gene amplicon data sets. Fifty-three samples included both types of data sets. An OTU table including all of the processed samples was obtained from HMP Qiime Community Profiling (23, 24), from which *H′* values per sample were estimated. Values were compared against *N*_*d*_ values derived from the metagenomic data sets by body site (medians per site) and by sample (intersection samples). Finally, we evaluated the resolution of *N*_*d*_ compared to *H′* by using set III, a collection of marine samples derived from two global sampling projects, 228 samples from the Tara Oceans expedition between September 2009 and March 2012 (12) and 64 samples from the Global Ocean Sampling expedition between March 2009 and December 2010 funded by the Beyster Family Fund and the Life Technology Foundation (SRA BioProject accession no. PRJEB10418). Sample metadata, as well as preprocessed reads and 16S rRNA gene OTU tables, were obtained from EBI Metagenomics (11). ANOVA was performed to evaluate the effects of different metadata variables on both *N*_*d*_ and Bayesian analysis-corrected *H′* of extracted 16S rRNA genes. The variables considered were size fraction (categorical), absolute value of latitude (i.e., degrees from the equator), latitude (degrees), geographic location (Mediterranean Sea, North Atlantic Ocean, North Pacific Ocean, Indian Ocean, Red Sea, South Atlantic Ocean, South Pacific Ocean, or Southern Sea), sampling date, and two decomposed seasonal components of sampling date. The decomposed seasonal components were estimated as the sine (vernality) and cosine (wintriness) of the date in radians (1 year = 2π) for samples in the Northern Hemisphere or the negative sine and negative cosine, respectively, for samples in the Southern Hemisphere.

10.1128/mSystems.00039-18.9 TABLE S3  Metagenomic samples from diverse environments used in this study. Download TABLE S3, PDF file, 0.1 MB. Copyright © 2018 Rodriguez-R et al. 2018 Rodriguez-R et al. This content is distributed under the terms of the Creative Commons Attribution 4.0 International license.

### Nonpareil 3 availability.

Nonpareil 3 is available for online analyses at http://enve-omics.ce.gatech.edu/nonpareil. The Nonpareil code is freely distributed under the artistic license 2.0 and is available at https://github.com/lmrodriguezr/nonpareil.
