# Supplementary figures and images for: Nonpareil 3: Fast Estimation of Metagenomic Coverage and Sequence Diversity
Source: mSystems. 2018 Apr 10;3(3):e00039-18. doi: 10.1128/mSystems.00039-18 (PMC5893860; doi:10.1128/mSystems.00039-18)

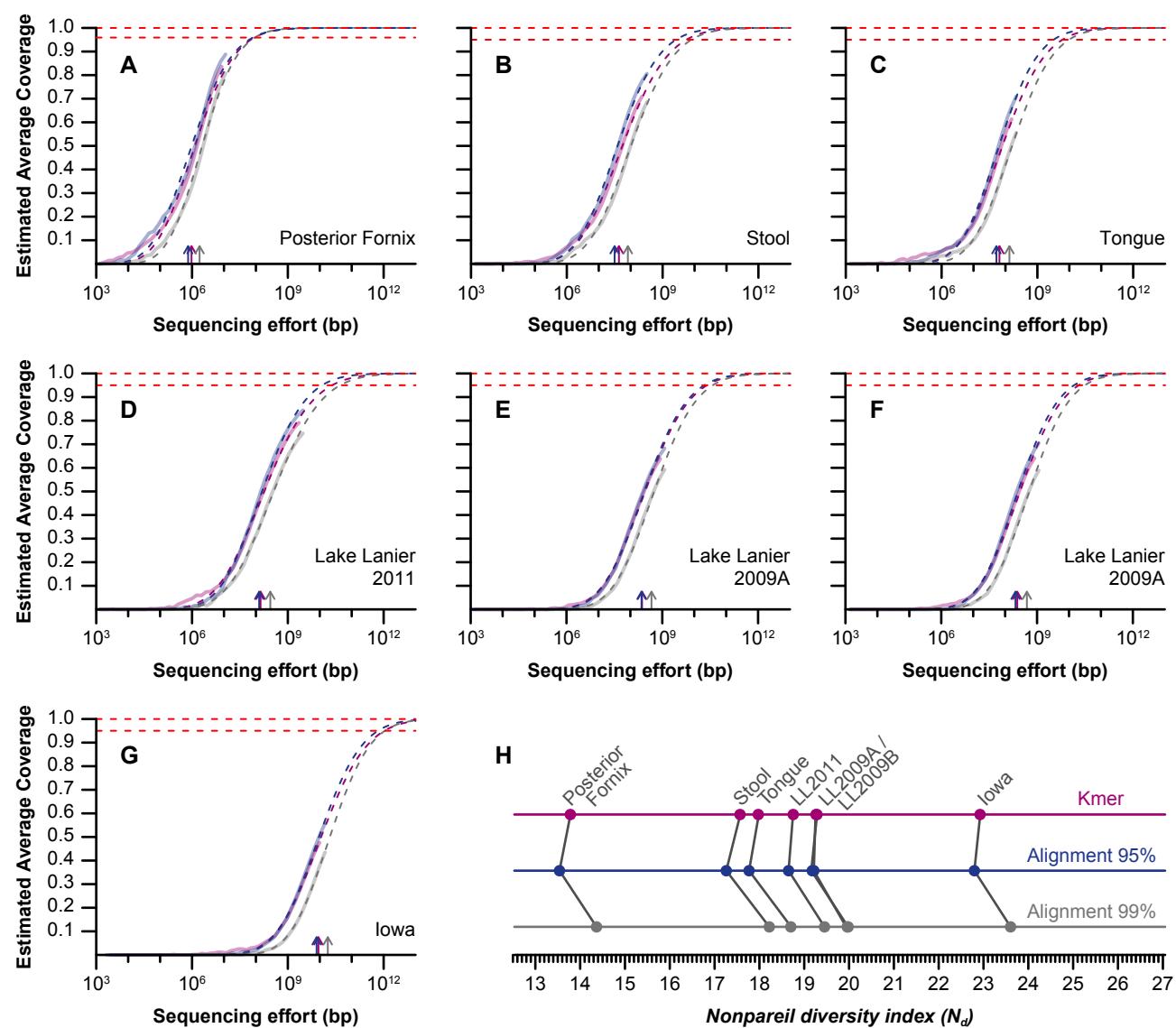

Supplement: FIG S1 [file sys003182225sf1.pdf]

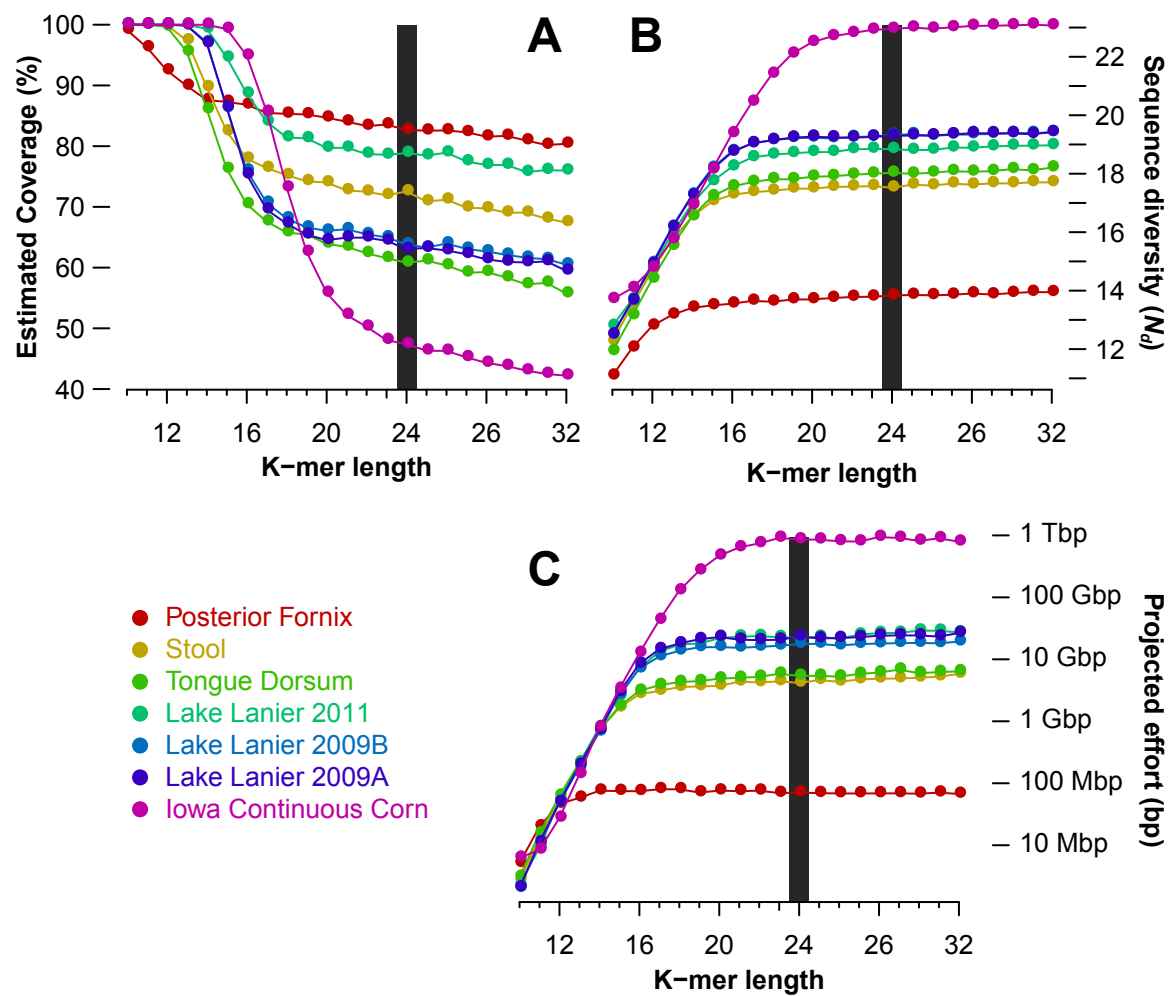

Supplement: FIG S2 [file sys003182225sf2.pdf]

**K-mer kernel**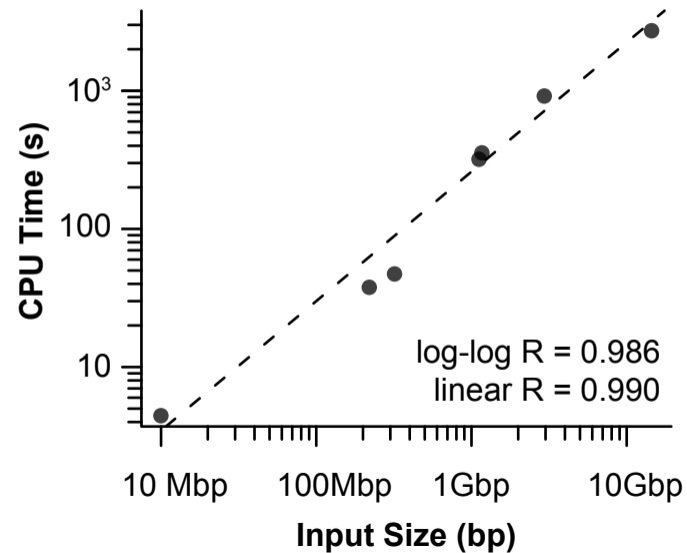**Alignment kernel**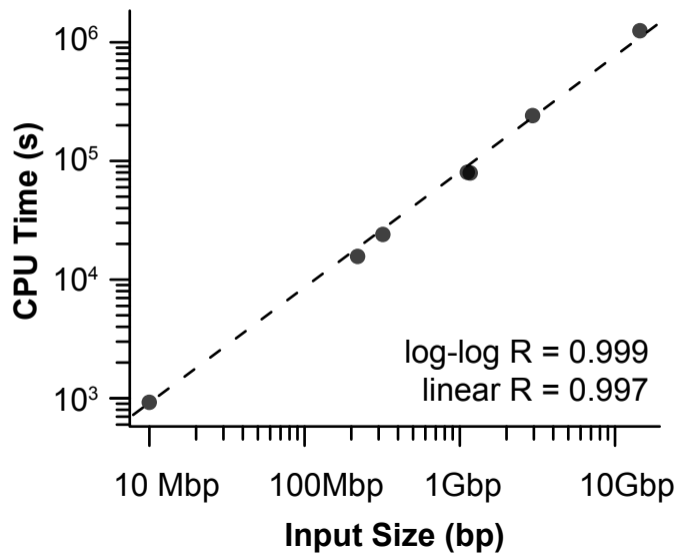

Supplement: FIG S3 [file sys003182225sf3.pdf]

### A. Low coverage

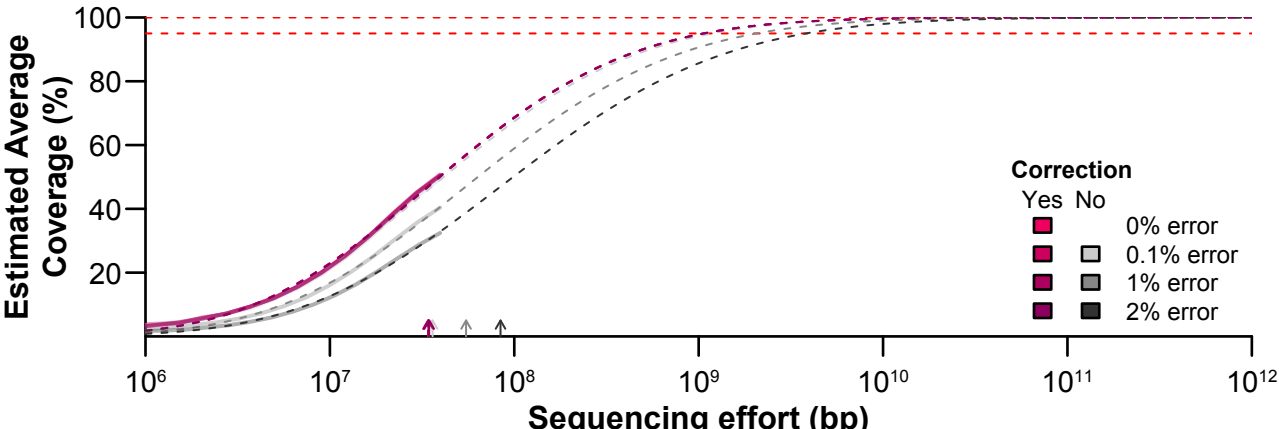

### B. High coverage

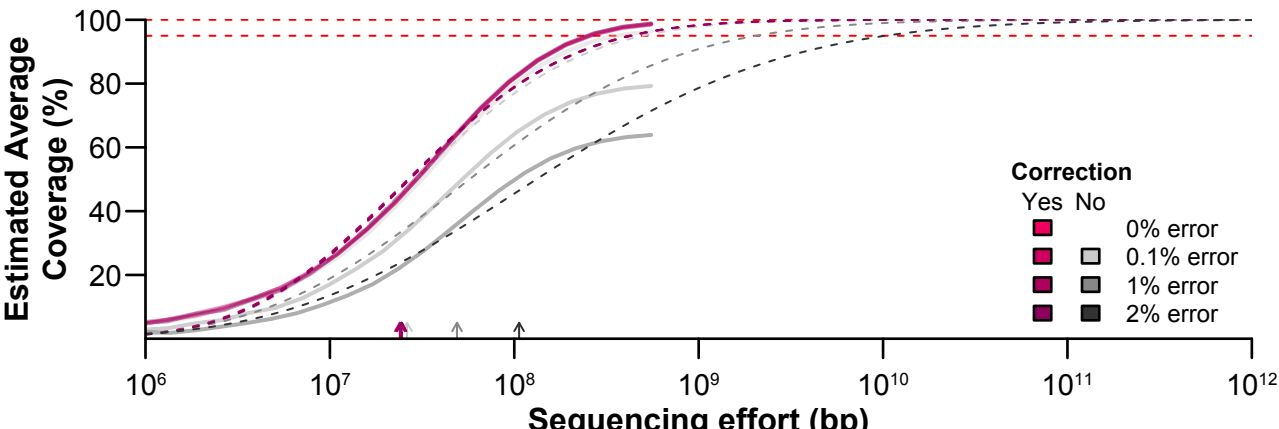

Supplement: FIG S4 [file sys003182225sf4.pdf]

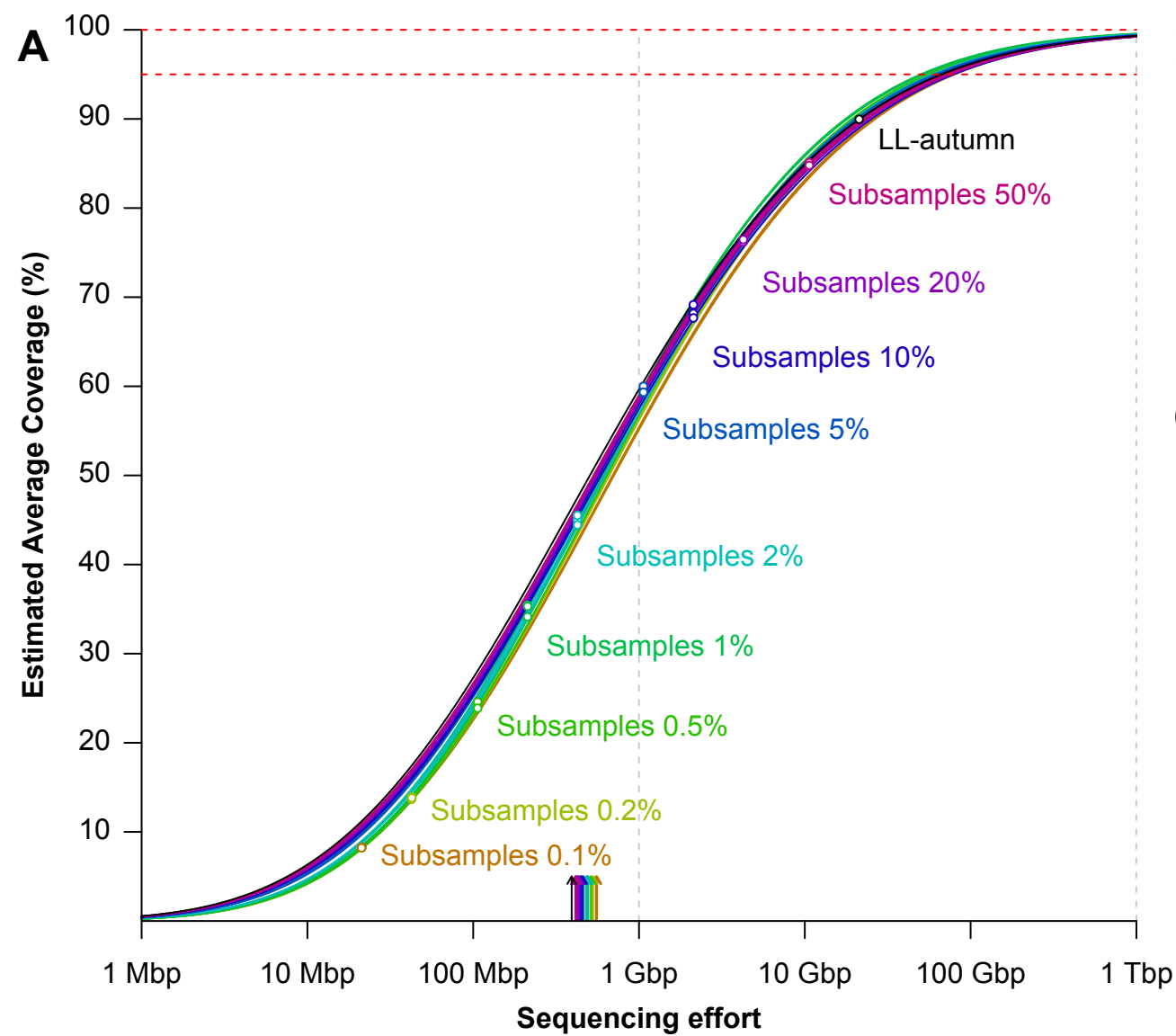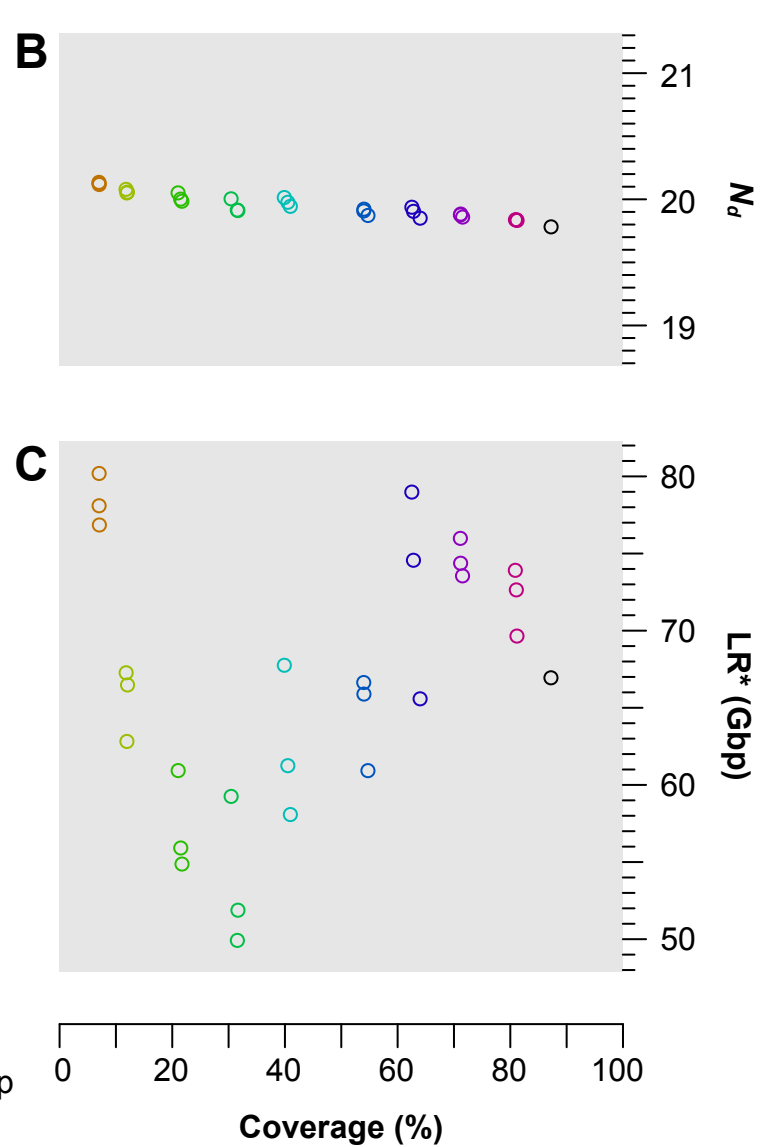

Supplement: FIG S5 [file sys003182225sf5.pdf]

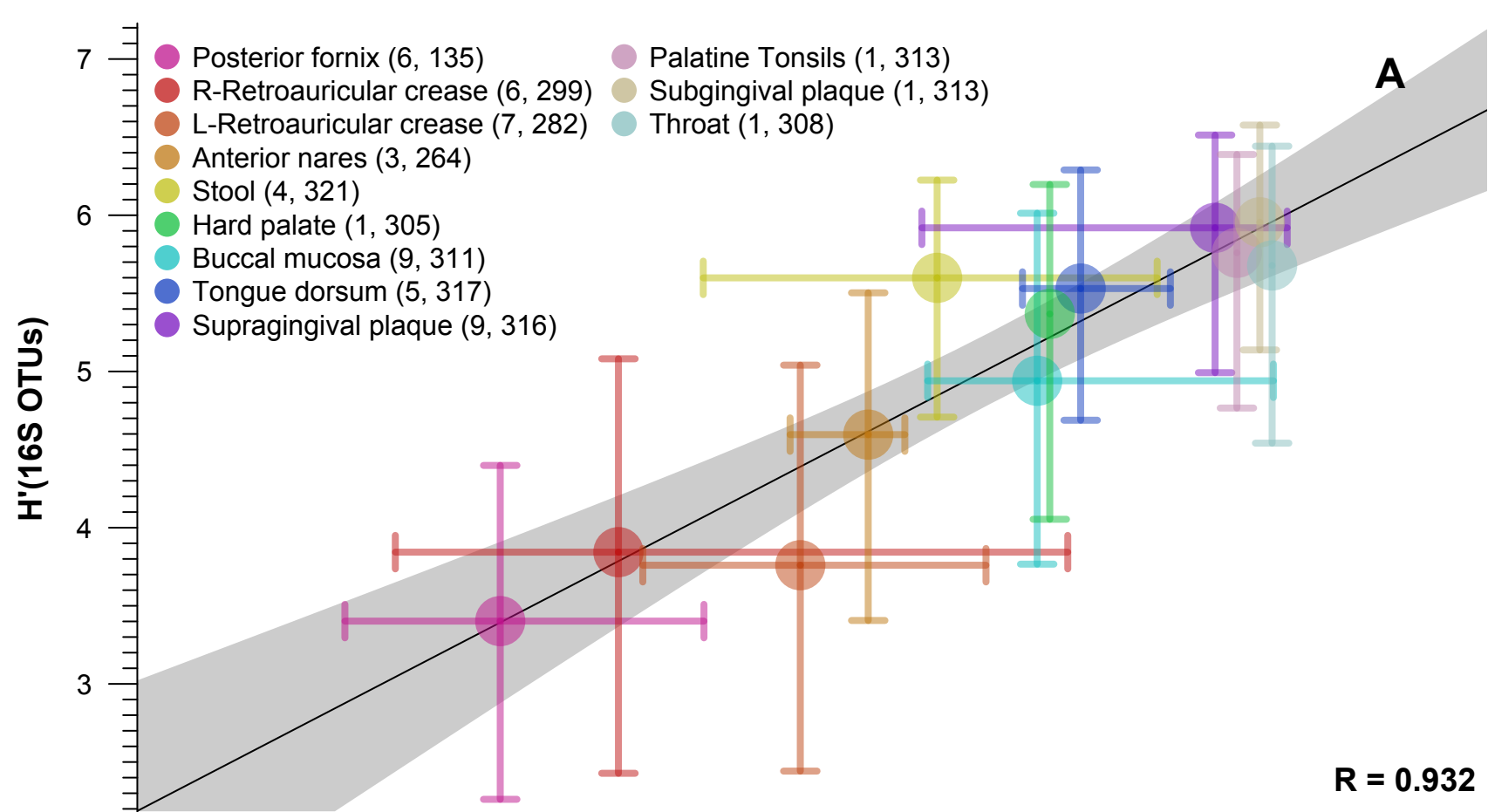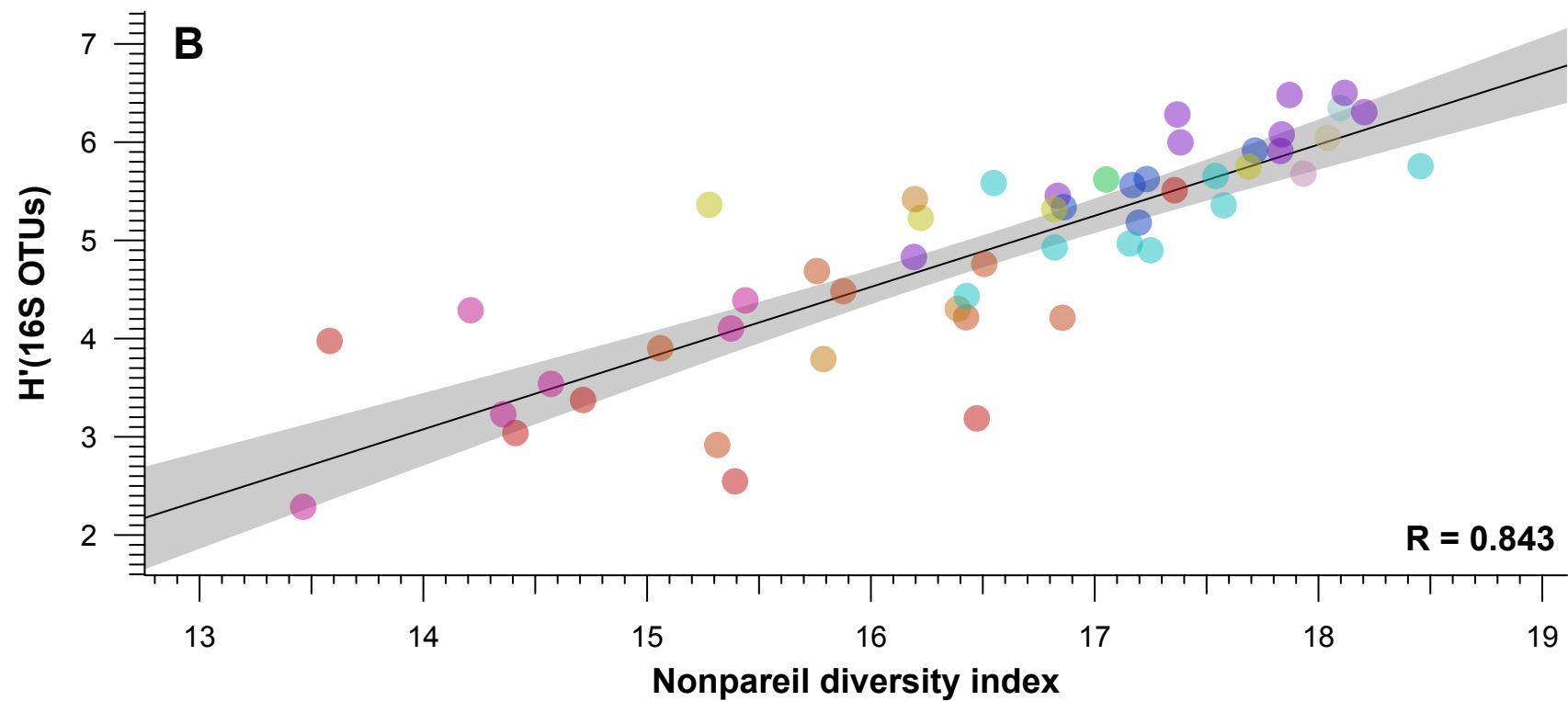

Supplement: FIG S6 [file sys003182225sf6.pdf]
