# Supplementary material for: Nonpareil 3: Fast Estimation of Metagenomic Coverage and Sequence Diversity
Source: mSystems. 2018 Apr 10;3(3):e00039-18. doi: 10.1128/mSystems.00039-18 (PMC5893860; doi:10.1128/mSystems.00039-18)
Supplement: TABLE S1 [file sys003182225st1.pdf]

Supplementary Table S1. Genomes used for simulated datasets to evaluate error correction.

Entry	Size (bp)	Abundance (%)[1]
NC_015189.1	5,178	6.33
NC_010940.1	3,533	1.00
NC_010580.1	181,736	1.78
NC_017867.1	1,938,822	2.71
NC_015052.1	2,400,312	0.45
NC_015920.1	29,448	1.02
NC_009713.1	3,678	4.58
NC_015914.1	6,221,273	4.95
NC_008255.1	4,433,218	0.06
NC_011661.1	1,855,560	0.54
NC_017910.1	4,158,725	2.20
NC_017631.1	5,131,397	5.55
NC_011419.1	100,021	1.27
NC_017642.1	90,868	1.75
NC_014558.2	53560	10.8
NC_013199.1	2968598	5.25
NC_008576.1	4719581	1.51
NC_014002.1	2012424	1.57
NC_010727.1	25164	0.54
NC_014563.1	165693	7.18
NC_016818.1	4861101	2.56
NC_007908.1	4712337	2.84
NC_015729.1	63532	0.90
NC_007954.1	4545906	4.43

NC_010506.1	5935403	5.19
NC_014009.1	5398	10.0
NC_017098.1	3285855	1.00
NC_015757.1	3551206	4.74
NC_017268.1	1139281	1.69
NC_016842.1	1139330	5.58

[1] Relative abundance of reads.
