# Supplementary material for: Nonpareil 3: Fast Estimation of Metagenomic Coverage and Sequence Diversity
Source: mSystems. 2018 Apr 10;3(3):e00039-18. doi: 10.1128/mSystems.00039-18 (PMC5893860; doi:10.1128/mSystems.00039-18)
Supplement: TABLE S2 [file sys003182225st2.pdf]

Supplementary Table S2. Nonpareil error correction for *k*-mer kernel.

**Error Rate**	**Coverage (%)**		**Req. effort (Gbp)**	
	**On[1]**	**Off[2]**	**On[1]**	**Off[2]**
**0.0%**	**51**	**51**	**1.06**	**1.06**
0.1%	51	49	1.06	1.18
1.0%	50	40	1.07	2.06
2.0%	50	32	1.07	3.82
**0.0%**	**99**	**99**	**0.43**	**0.43**
0.1%	98	96	0.43	0.50
1.0%	99	79	0.43	2.05
2.0%	99	64	0.42	10.3

[1] Nonpareil with *k*-mer kernel and error correction.

[2] Nonpareil with *k*-mer kernel and without error correction.
