# Supplementary material for: Nonpareil 3: Fast Estimation of Metagenomic Coverage and Sequence Diversity
Source: mSystems. 2018 Apr 10;3(3):e00039-18. doi: 10.1128/mSystems.00039-18 (PMC5893860; doi:10.1128/mSystems.00039-18)
Supplement: TABLE S3 [file sys003182225st3.pdf]

Supplementary Table S3. Metagenomic samples from diverse environments used in this study.

Project[1]	Sample[1]	Run[1]	Platform	Biome / Sample Name
				**Animal Host**
ERP014522	ERS1079001	ERR1307052	Illumina	Broiler Caecum
ERP005429	ERS428046	ERR469647	Ion Torrent	Gir Cow Rumen
SRP056641	SRS971427	SRR2240256	Illumina	Human Anterior nares
SRP056641	SRS1055060	SRR2241209	Illumina	Human Buccal mucosa
ERP005507	ERS430636	ERR472949	Illumina	Human Medieval Skeleton
ERP006678	ERS537696	ERR589719	Illumina	Human Rheumatoid Gut
ERP011339	ERS805755	ERR981247	Illumina	Human Skin
SRP056641	SRS1041031	SRR2175645	Illumina	Human Stool
SRP056641	SRS893385	SRR1952623	Illumina	Human Supragingival plaque
SRP056641	SRS893352	SRR1952591	Illumina	Human Tongue dorsum
ERP005462	ERS428192	ERR470082	Ion Torrent	Kankrej Cow Rumen
ERP020572	ERS1474381	ERR1762125	Illumina	Mouse Colitis Stool
ERP006800	ERS569458	ERR658091	Illumina	Mouse DarkPhase Stool
ERP020572	ERS1474383	ERR1762111	Illumina	Mouse Stool
ERP015203	ERS1122653	ERR1367842	Illumina	Mouse Stool
ERP013165	ERS970408	ERR1135432	Illumina	Pig Stool
ERP013942	ERS1037701	ERR1223843	Illumina	Pig Stool B
ERP021211	ERS1566221	ERR1854461	Illumina	Salmon Faeces
				**Engineered**
ERP011345	ERS805497	ERR977414	Illumina	Austria Petrochemical Waste
SRP046072	SRS693743	SRR1562011	Illumina	China Liquor 20y
SRP046072	SRS693731	SRR1561999	Illumina	China Liquor 220y

SRP046072	SRS693721	SRR1561989	Illumina	China Liquor 440y
SRP046072	SRS693735	SRR1562003	Illumina	China Liquor 50y
ERP009124	ERS632930	ERR712390	Illumina	Danish Activated Sludge
ERP011345	ERS805491	ERR977425	Illumina	Danish Petrochemical Waste
ERP015410	ERS1215587	ERR1467155	Illumina	Danish Wastewater
ERP005835	ERS627438	ERR699788	Illumina	France Cheese
ERP011345	ERS805499	ERR977420	Illumina	German Petrochemical Waste
ERP014522	ERS1078367	ERR1306535	Illumina	Italy Broiler Meat
SRP003198	SRS211744	SRR256754	454	Kimchi
ERP011345	ERS805494	ERR977405	Illumina	Russian Petrochemical Waste
ERP021211	ERS1545971	ERR1823566	Illumina	Salmon Feed
ERP015657	ERS1162552	ERR1414231	Illumina	Swedish Activated Sludge
ERP015657	ERS1162594	ERR1414273	Illumina	Swedish Primary Sludge
ERP015657	ERS1162544	ERR1414223	Illumina	Swedish Wastewater
				**Enrichment**
ERP016191	ERS1276023	ERR1554596	Illumina	*Achromatium* populations from Lake Stechlin
ERP010072	ERS700853	ERR845264	Illumina	Methanesulfonate Enrichment
SRP013339	SRS334564	SRR500503	Illumina	Naphtalene Enrichment
ERP017906	ERS1408748	ERR1688863	Illumina	Oil Enrichment
				**Freshwater**
ERP013793	ERS1030529	ERR1201173	Illumina	Alpine Glacier
ERP006615	ERS524244	ERR575705	Illumina	Australia Bilabong Uranyl
ERP016382	ERS1245396	ERR1527265	Illumina	Australia Groundwater Radioactive
ERP021244	ERS1532239	ERR1816708	Ion Torrent	Curua Una River

ERP016063	ERS1443742	ERR1725848	Illumina	Daisy Lake
ERP021394	ERS1546685	ERR1824222	454	Diamante River
ERP009117	ERS632771	ERR711863	Illumina	Dianchi Lake
SRP080009	SRS1589004	SRR3960573	Illumina	Lake Mendota
ERP013741	ERS1027341	ERR1198911	Illumina	Movile Cave Water
ERP016482	ERS1261403	ERR1544444	Illumina	Nakdong River
SRP000240	SRS000608	SRR002326,SRR002327,SRR002328	454	Northern Schneeferner Glacier
ERP013793	ERS1030534	ERR1201178	Illumina	Pakistan Glacier
ERP013033	ERS950426	ERR1104488	Illumina	Rabindra Sarovar Lake
ERP012868	ERS956539	ERR1121555	Ion Torrent	Svalbard Glacier
ERP020465	ERS1467323	ERR1750013	Ion Torrent	Tucurui Lake
ERP016258	ERS1222671	ERR1474558	Illumina	Xiangxi River
				**Marine**
ERP001736	ERS487936	ERR598950	Illumina	Alboran Sea
SRP039390	SRS581965	SRR1204581	Illumina	Amazon Plume
ERP001736	ERS489074	ERR599053	Illumina	Arabian Sea
ERP004692	ERS399638	ERR420363, ERR420364, ERR420365, ERR420366, ERR420367, ERR420368	Illumina	Axial Seamount Hydrothermal Vent
ERP018210	ERS1418479	ERR1698987	Illumina	Copano Bay
ERP018210	ERS1418469	ERR1698980	Illumina	Galveston Bay
ERP001736	ERS489315	ERR599138	Illumina	Indian Sea

ERP001736	ERS488509	ERR599094	Illumina	Mediterranean Sea
ERP017354	ERS1370021	ERR1662525	Illumina	MidCayman Rise Subseafloor
ERP008814	ERS612887	ERR688354	Illumina	Noosa Estuary
ERP001736	ERS494374	ERR598958	Illumina	North Atlantic Ocean
ERP001736	ERS493372	ERR598980	Illumina	North Pacific Ocean
ERP018210	ERS1418484	ERR1698991	Illumina	Nueces Bay
ERP001736	ERS488545	ERR599106	Illumina	Red Sea
ERP001736	ERS490433	ERR598984	Illumina	South Atlantic Ocean
ERP001736	ERS491804	ERR599024	Illumina	South Pacific Ocean
				**Soil**
SRP039575	SRS567760	SRR1185960	Illumina	Arsenic Antimony Soil
ERP008551	ERS580791	ERR671919	Illumina	Australian Calcarosols
ERP004852	ERS402379	ERR430975	Ion Torrent	Australian Forest
ERP008551	ERS580784	ERR671939	Illumina	Australian Tenosols
ERP008551	ERS580794	ERR671913	Illumina	Australian Vertosols
ERP014473	ERS1075580	ERR1303295	Illumina	Florianopolis Dune
ERP014473	ERS1075581	ERR1303296	Illumina	Florianopolis Forest
SRP046227	SRS697016	SRR1569466	Illumina	Florida Beach Sediments
SRP017930	SRS386691	SRR650839	Illumina	Konjac Plowland
SRP033484	SRS510679	SRR1042602	Ion Torrent	Kutch Saline Desert Soil
SRP029969	SRS481052	SRR988096	Illumina	Oklahoma Grassland
SRP018858	SRS398270	SRR770299	Illumina	Swamp
SRP008617	SRS265882	SRP008617	Illumina	Wisconsin Corn Soil
SRP008628	SRS265900	SRP008628	Illumina	Wisconsin Native Prairie
SRP008595	SRS265861	SRP008595	Illumina	Wisconsin Switchgrass Soil
				**Mock**

PRJNA48475	SAMN00120089	SRR172903[2]	Illumina	HMP Mock Staggered
PRJNA48475	SAMN00120089	SRR072232[2]	454	HMP Mock Staggered
PRJNA48475	SAMN00120088	SRR172902[2]	Illumina	HMP Mock Even
PRJNA48475	SAMN00120088	SRR072233[2]	454	HMP Mock Even
PRJNA324704	SAMN05213576	SRR3656745[2]	Illumina	MBARC-26
NA[3]	NA[3]	NA[3]	Illumina	Mock M63H 25M

[1] Identifiers in EBI Metagenomics, derived from the EBI European Nucleotide Archive (ENA) or the NCBI Sequence Read Archive (SRA).

[2] Not available in EBI Metagenomics, diversity directly estimated from theoretical concentrations.

[3] This dataset was obtained from https://mgexamples.s3.climb.ac.uk/MOCK_M63H.25M.1.fastq.gz.
